# Experiences of discrimination are associated with microbiome and transcriptome alterations in the gut

**DOI:** 10.3389/fmicb.2024.1457028

**Published:** 2024-10-24

**Authors:** Tien S. Dong, Simer Shera, Kirstin Peters, Gilbert C. Gee, Hiram Beltrán-Sánchez, May C. Wang, Lisa A. Kilpatrick, Xiaobei Zhang, Jennifer S. Labus, Allison Vaughan, Arpana Church

**Affiliations:** ^1^G. Oppenheimer Center for Neurobiology of Stress and Resilience at UCLA, Los Angeles, CA, United States; ^2^UCLA Vatche and Tamar Manoukian Division of Digestive Diseases, Los Angeles, CA, United States; ^3^David Geffen School of Medicine at UCLA, Los Angeles, CA, United States; ^4^Goodman Luskin Microbiome Center at UCLA, Los Angeles, CA, United States; ^5^Department of Medicine, University of California, Los Angeles, Los Angeles, CA, United States; ^6^Department of Community Health Sciences Fielding School of Public Health, University of California, Los Angeles, Los Angeles, CA, United States; ^7^California Center for Population Research, UCLA, Los Angeles, CA, United States

**Keywords:** discrimination, psychosocial stressor, gut microbiome, *Prevotella*, transcriptome

## Abstract

**Background:**

Discrimination is a recognized psychosocial stressor that has been linked to various negative health outcomes. This study explored the impact of discrimination on gut health, specifically focusing on microbiome changes, predicted metagenomic differences, transcriptomic profiles, and the potential for using a multi-omic approach to predict discrimination to identify discrimination status for an individual. Methods: We conducted a comprehensive investigation involving male and premenopausal female participants, using the Everyday Discrimination Scale to classify them into either high or low discrimination. Multiple questionnaires were administered to evaluate participants’ physiological, psychological, and perceived stressors. Two diet questionnaires were also administered. Stool samples were collected for microbiome analysis and RNA sequencing. Microbial composition changes were analyzed using the Shannon index and Chao1 richness estimator for alpha diversity and the Aitchison distance metric for beta diversity. Differential abundance was evaluated using MaAsLin2, followed by metatranscriptomics sequencing and annotation. A multi-omic approach utilizing random forest was used to assess the predictability of discrimination.

**Results:**

The study results showed that high discrimination was linked to higher gut microbiome species richness (Chao1, *p* = 0.02) and significant beta diversity differences (*p* = 0.04). Prevotella and Ruminococcaceae were both less abundant in the high discrimination group. High discrimination participants also reported higher levels of depression, anxiety, perceived stress, early life adversity, visceral sensitivity, and neuroticism than those in the low discrimination group. Gene expression analysis revealed distinctive patterns, with significant changes in genes associated with environmental sensing (two-component system) and metabolic pathways. In a plot comparing gene transcription to DNA content, certain genes showed higher expression levels in participants who experienced both high and low levels of discrimination. Our random forest classifier demonstrated the capability to accurately differentiate individuals with high and low discrimination in our training cohort (AUC = 0.91).

**Conclusion:**

These findings illuminate the substantial impact of discrimination on gut health, encompassing microbiome composition, gene expression, and functional pathways. These findings suggest that discrimination is associated with internal biological changes that can be associated with negative health outcomes, opening research to examine novel pathways that can be used to mitigate the negative health effects of discrimination.

## Introduction

Discrimination, or the differential treatment of individuals based on existing or perceived membership to a specific identity, plays a significant role in the social structures that perpetuate inequality and injustices (Schmitt and Branscombe, 2002; Slopen and Williams, 2014). The role of discrimination and one’s place in either an “advantaged” or “disadvantaged” group has clear implications for their prospective healthcare outcomes (Berger and Sarnyai, 2015). However, the biological pathways by which discrimination impacts mental and physical health remain unclear.

Previous research has consistently revealed that belonging to disadvantaged groups is directly associated with poorer health outcomes (Melchior et al., 2007; Banerjee, 2016; Killen et al., 2016). Several pathways have been described to link discrimination to health, with the most popular being the stress response provoked by discrimination via the activation of the hypothalamic–pituitary–adrenal (HPA) axis. Physical arousal that results from the experience of discrimination can activate the HPA axis, resulting in the release of cortisol (Keller et al., 2017). Cortisol release as a result of dysregulation of the HPA axis has been implicated in the pathophysiology of anxiety and depression (Tetel et al., 2018; Lyte et al., 2020; Foster et al., 2017).

Recent studies, however, have pushed for an investigation of the gut microbiome in influencing the psychosocial stress response via the gut–brain axis as well (De Palma et al., 2015; Moussaoui et al., 2017). The gut microbiome and the brain communicate through various pathways, such as the HPA axis and the immune system (Dong et al., 2023; Cryan et al., 2019; Marcondes Ávila et al., 2020). Stress can dysregulate these pathways, interrupting the communication between the gut and the brain. It is well documented that stress-induced changes to the brain can result in a compromised brain–gut signaling network, leading to alterations in the gut microbiome, increased intestinal permeability, microbial dysbiosis, and changes to microbial gene expression (Keita and Söderholm, 2010; Moreira et al., 2016; Ritter et al., 2012; Vetter et al., 2010; Higginson and McNamara, 2016; Van Dyken and Lacoste, 2018). These changes can subsequently result in immune system activation and thus an inflammatory response by the host (Sarubbo et al., 2022; Chakrabarti et al., 2022). Despite emerging evidence linking stress, the gut–brain axis, and health outcomes, there is still a significant gap in understanding how discrimination specifically impacts these biological pathways. Understanding these effects is crucial in revealing novel biomarkers of discrimination-related stress and providing insights into targeted interventions to mitigate adverse health impacts.

The purpose of this study was to understand the exact impact of discrimination on the gut microbiome and its correlation with healthcare outcomes. To comprehensively understand how discrimination can affect biological pathways linked to outcomes in health, we performed a detailed analysis of the effects of discrimination on the gut microbiome in a racially diverse population, specifically focusing on changes to the gut microbiome composition, predicted metagenome differences, and transcriptomic profiles. These alterations were also examined in correlation with health outcomes such as depression, anxiety, and perceived stress. Using these results, we evaluated the potential for using a random forest classifier to predict discrimination to identify discrimination status for an individual.

## Materials and methods

### Ethics approval and consent to participate

The procedures performed were approved by the Institutional Review Board at the University of California, Los Angeles, Office of Protection for Research Subjects. All participants provided written informed consent.

### Study cohort

Individuals were recruited from clinics and communities in Los Angeles, California. To be eligible for the study, participants had to be at least 18 years old. We excluded individuals who took antibiotics or probiotics in the 3 months preceding the study. Because this part of the study was part of a larger study involving magnetic resonance scanning, participants were also excluded if they used analgesic drugs or medications that interfered with the central nervous system, weighed over 400 pounds (due to magnetic resonance imaging scanning weight limits), or were lefthanded (since there is variability in brain scans due to hand dominance). The initial cohort consisted of 165 adults, and the final sample consisted of 154 adults. Participant data included the following: blood samples for genetic expression, stool samples for microbial and transcriptomic analysis, anthropometrics, diet history, and verified medical survey questionnaires. Participants self-reported race/ethnicity (Asian American, Black, Hispanic, or White). For female participants, only premenopausal individuals were enrolled, and biological samples were collected in the follicular phase of their menstruation cycle, as determined by the reported date of their last menstrual period. This approach aimed to minimize any hormonal influence on the biological and microbiome samples.

### Questionnaires

The following validated questionnaires were used to assess the cohort members: Everyday Discrimination Scale (EDS), Connor Davidson Resilience Scale (CD-RISC), Early Traumatic Inventory (ETI), Hospital Anxiety and Depression (HAD) scale, International Personality Item Pool (IPIP), Perceived Stress Scale (PSS), State–Trait Anxiety Inventory (STAI), Visceral Sensitivity Index (VSI), Socioeconomic Status (SES), Patient Health Questionnaire-15 (PHQ-15), and Short Form Health Survey (SF12). All participants also participated in a Diet History Questionnaire (DHQ) III and the UCLA Diet Checklist (Lenhart et al., 2022).

EDS was used to assess discrimination. It measures routine and chronic experiences of unjust treatment, categorizing subjects into a high EDS level or a low EDS (Michaels et al., 2019). The questionnaire has been widely used in diverse populations, consisting of nine-item questions to assess how frequently someone perceives discrimination in their day-to-day life with frequency rate options of ‘never,’ ‘less than once a year,’ ‘a few times a year,’ ‘a few times a month,’ ‘at least once a week,’ and ‘almost every day.’ These answers are assigned a score on a Likert scale, with ‘never’ equating to zero and ‘almost every day’ equating to a five and a maximum score of 50. Follow-up questions are asked, querying the responder to speculate on the main reason for these experiences if they answered a few times a year or more frequently to any initial questions.

The CD-RISC, ETI, HAD, IPIP, PSS, STAI, and VSI were used as psychological and symptom instruments. The CD-RISC was used to measure stress-coping ability by assessing resilience (Connor and Davidson, 2003). It includes 25 items, each scored on a scale from 0 to 4, with higher scores reflecting greater resilience in respondents. The ETI was used to measure self-reported childhood trauma, including questions from general, physical, emotional, and sexual trauma, as well as additional questions for serious trauma (Jeon et al., 2012). The HAD scale assessed anxiety and depression symptoms in the participants (Edelstein et al., 2010). Respondents were asked to rate 14 different items on a 4-point severity scale, producing results for both anxiety and depression. The IPIP was used to assess personality, categorizing an individual into the big five personality traits (Ypofanti et al., 2015). The PSS was used to measure one’s perception of stress based on their evaluation of the degree to which specific situations are categorized as stressful (Nater and Ali, 2020). The STAI was used to assess anxiety in cohort members (Carmin and Ownby, 2010). The questionnaire includes four separate dimensions of “state” and “trait” anxiety, including feelings of tension, worry, and more. The VSI was used to measure gastrointestinal symptom-specific anxiety (Trieschmann et al., 2022). The questionnaire consists of 15 items ranked on the same Likert scale described previously, with examples of items including statements such as “I worry that abdominal pain might be due to a serious illness” and “I am afraid that when I experience stomach discomfort, it will get worse.”

The SF12 was used to assess an individual’s quality of life by quantifying the impact of health on daily life (Huo et al., 2018). Both the DHQ-III and UCLA Diet Checklist were used for the evaluation of participants’ diets.

### Statistical analyses

Differences in baseline demographic characteristics were assessed using Student’s *t*-test for continuous variables and the chi-squared test for categorical variables. For the purposes of our analyses, we dichotomized EDS to either “high discrimination” (EDI > =6) or “low discrimination” (EDI < 6) using the median score of our study population. This process determined a cutoff using the study population similar to previously published studies (Chang et al., 2018).

Changes to microbial composition were analyzed using several metrics. Raw microbiome reads were processed through DADA2 (Callahan et al., 2016). The microbiome dataset was rarefied to a standardized sequence depth of 34,222 reads. Alpha diversity—the diversity within a sample—was computed using the Shannon (a measure of species evenness) and Chao1 (a measure of species richness) indices through QIIME2. The statistical significance of the Shannon and Chao1 indices was determined using analysis of variance (ANOVA) using the aov function in R. Beta diversity—the variability in composition among the community—was analyzed using the robust Aitchison distance metric within the DEICODE package of QIIME2 (Sawyer et al., 2012). Significance in beta diversity was evaluated using permutational multivariate analysis of variance, specifically employing the ‘adonis’ package in R (Version 4.1.2), adjusting for sex, age, body mass index (BMI), and diet. Differential abundance analysis of genera was computed using MaAsLin2 (Mallick et al., 2021).

Fecal samples were sent to Viome Life Sciences, Inc., for RNA extraction, metatranscriptomics sequencing, and annotation (Hatch et al., 2019). Functional annotation of genes using metatranscriptomics allowed for the identification and comparison of genes expressed by the high and low discrimination groups. RNA extraction was performed via bead beating; DNase was used to degrade DNA, and subtractive hybridization was used to deplete 16S/23S ribosomal RNA. The resulting RNA was used to prepare sequencing libraries. The libraries were processed through 150×2 paired-end sequencing on Illumina NovaSeq. Sequence reads were aligned to a precomputed unique k-mers database. To perform functional annotation, the sequence reads were aligned to the MetaHIT Consortium integrated gene catalog (MetaHIT Consortium, 2014).

PICRUst2, a reputable tool used to attain metagenomic data from 16S rRNA compositional data, was used to infer metagenomic data from the 16S rRNA sequencing data of each sample. The 16S rRNA sequencing data were inputted into the PICRUst2 program and normalized by copy number. Default parameters were used. The Kyoto Encyclopedia of Genes and Genomes (KEGG) database was used to categorize subsequent metagenes by function. DESeq2 was used to identify differences in predicted metagenes by discrimination, with *p*-values adjusted for multiple hypothesis testing.

Using a random forest classifier, a model incorporating 16S data, transcriptomic data, and metagenomic data was created using the variables that had at least a p-value of <0.1 to classify participants as either high or low discrimination. Random forest analysis was conducted utilizing the `randomForest` package in R. The analysis was executed with default settings, including a forest size of 1,000 trees. To assess the model’s performance and generalization ability, k-fold cross-validation was employed, with k set to a value of 10. This procedure ensures robustness and minimizes overfitting by partitioning the dataset into k equally sized folds, where each fold serves as a validation set while the remaining data are utilized for training. The process is repeated k times, with each fold serving once as the validation set. This approach allows for an unbiased estimation of the model’s predictive performance.

Clinical measures as evaluated by the abovementioned questionnaires and demographic data were correlated with discrimination using analysis of variance for continuous variables and the chi-square tests for categorical variables.

## Results

### Participant characteristics

Of the 154 participants, 141 submitted fecal samples for analysis. The participants consisted of 30 Asian individuals (13 high EDS and 17 low EDS), 18 Black individuals (11 high EDS and 7 low EDS), 57 Hispanic individuals (32 high EDS and 25 low EDS), and 36 White individuals (19 high EDS and 17 low EDS) (Table 1).

**Table 1:** Participant characteristics and clinical questionnaires.

	Low EDS (*n* = 74)	High EDS (*n* = 80)	*P*-value
Mean EDS (SD)	2.1 (2.1)	12.9 (5.7)	**<0.001**
Male participants (*n* = 43)	44.20%	55.80%	0.55
Female participants (*n* = 111)	49.50%	50.50%	
Age (year) (SD)	32.6 (10.3)	30.5 (10.3)	0.21
BMI (SD)	29.9 (5.9)	29.9 (5.8)	0.98
Education			
Some high school	0.4	0.6	0.88
High school graduate	47.30%	52.70%	
College graduate or higher	47.90%	52.10%	
Marital status			
Never married	43.90%	56.10%	0.39
Married	51.30%	48.70%	
Divorced	56.30%	43.80%	
Widowed	100.00%	0.00%	
Questionnaire scores (reference range: cutoff value)			
ETI total score (SD) (0–69; >16)	3.6 (4.3)	5.5 (4.4)	**0.009**
CDRISC score (SD) (0–100; NR)	80.5 (12.0)	76.9 (13.3)	0.09
IPIP neuroticism (SD) (0–240: NR)	20.1 (6.3)	23.1 (7.6)	**0.01**
IPIP extraversion (SD) (0–240; NR)	36.1 (7.0)	34.3 (7.1)	0.12
SES score (SD) (0–10; NR)	6.3 (1.4)	5.8 (1.5)	0.06
PHQ-15 score (SD) (0–30; min <5, low 5–9, medium 10–14, high >14)	4.5 (3.8)	5.9 (4.2)	**0.03**
STAI trait score (SD) (0–80; >37)	30.9 (7.5)	35.6 (10.7)	**0.002**
HAD anxiety (SD) (0–21; >7)	4.2 (3.6)	5.9 (3.7)	**0.005**
HAD depression (SD) (0–21; >7)	1.8 (1.8)	2.9 (2.9)	**0.009**
PSS score (SD) (0–40; >13)	10.8 (5.7)	14.8 (6.4)	**0.001**
SF12 physical score (SD) (0–100; >50)	54.2 (3.1)	52.4 (5.6)	**0.02**
SF12 mental score (SD) (0–100; >50)	53.1 (6.3)	49.7 (9.4)	**0.01**
VSI score (SD) (0–75, >37)	7.2 (10.1)	15.4 (17.7)	**0.0006**
Ethnicity/Race			
African American (*n* = 20)	35.00%	65.00%	0.44
Asian (*n* = 31)	58.10%	41.90%	
Hispanic (*n* = 62)	46.80%	53.20%	
White (*n* = 40)	47.50%	52.50%	
Other (*n* = 1)	100.00%	0.00%	

EDS, Everyday Discrimination Scale; BMI, body mass index; SD, standard deviation; ETI, early traumatic inventory; CDRISC, Connor Davidson Resilience Scale; IPIP, international personality item pool; SES, socioeconomic status; PHQ, physical health questionnaire; STAI, Stair Trait Anxiety Inventory; HAD, Hospital Anxiety and Depression Scale; PSS, Perceived Stress Scale; SF12, short form healthy survey; VSI, visceral sensitivity index. Significant *p*-value < 0.05 (bolded). Cutoff values and reference ranges for each questionnaire are listed adjacent to each questionnaire. NR, no established reference ranges.

No significant differences were observed in the proportion of missing microbiome or metabolomics data between the groups (*p* = 0.20).

Of the 154 participants, 80 were classified as high discrimination and 74 were classified as low discrimination. Compared to the low discrimination group, the high discrimination group presented with higher levels of depression (*p* = 0.009), anxiety (p = 0.009), perceived stress (*p* = 0.001), early life adversity (p = 0.009), visceral sensitivity (*p* < 0.001), neuroticism (*p* = 0.01), and overall poorer scores for physical (*p* = 0.02) and mental (p = 0.01) health. No significant differences were observed in age, BMI, education, marital status, or diet between the high and the low discrimination groups.

### Discrimination introduces changes to microbiome composition

**Figure 1:**
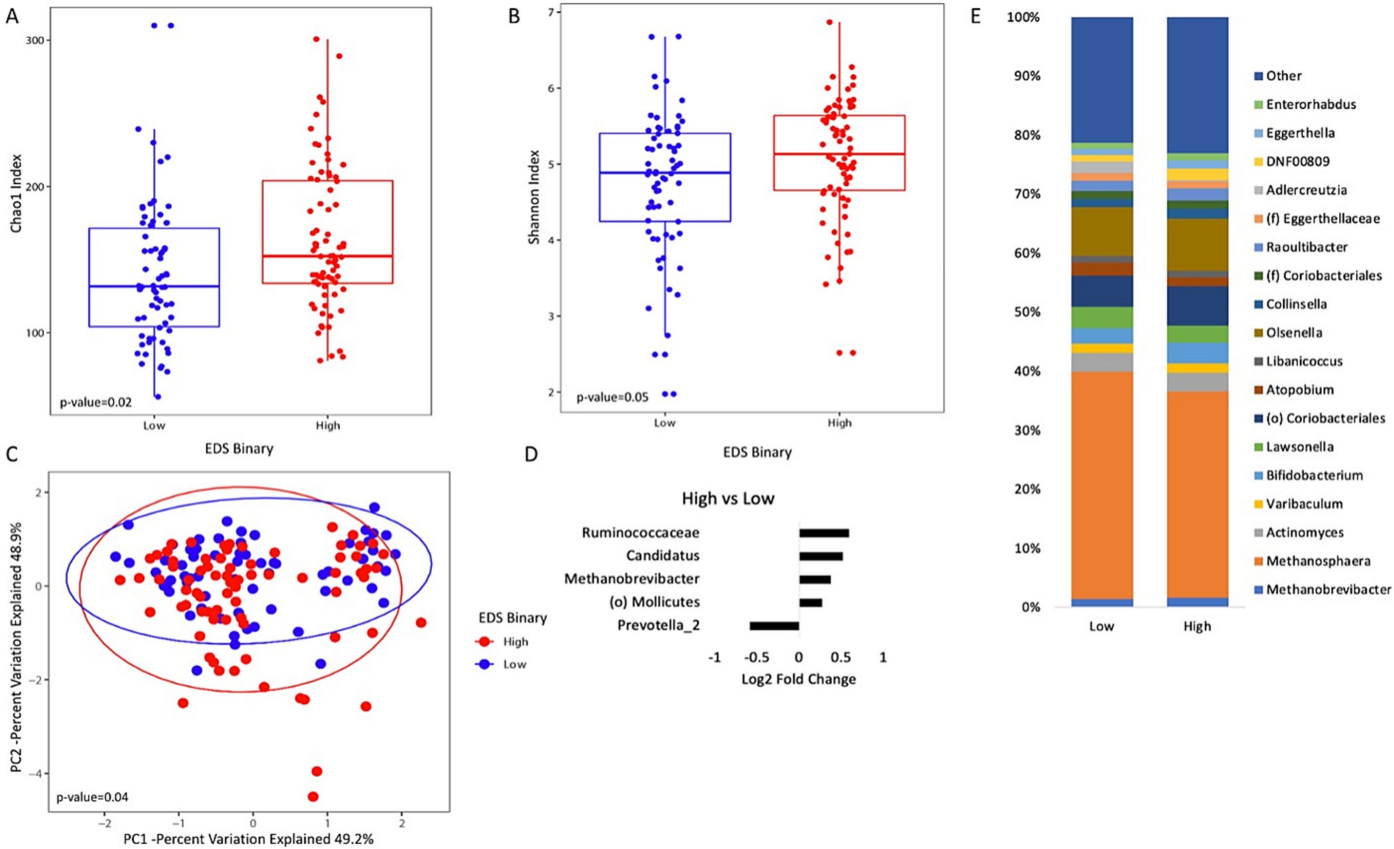
Analysis of gut microbiome diversity and taxonomic composition in the high and low discrimination groups. **(A)** Box plot of Chao1 index (species richness) and **(B)** Shannon index (species evenness). **(C)** Principal coordinate analysis plot of the microbiome composition by the high and low discrimination groups as measured by the Everyday Discrimination Scale (EDS). **(D)** MaAsLin2 output that shows which genera were differentially abundant in the high discrimination group compared to the low discrimination group. **(E)** Taxonomic plots of all genera by discrimination status. Only genera with a relative abundance of >1% are represented.

Analysis of the gut microbiome between the high and low discrimination groups indicates that discrimination is associated with clear microbiome changes. Both the Chao1 richness estimator and Shannon indices were used to measure alpha diversity for the high and low discrimination groups. Those with high levels of discrimination had higher levels of species richness (Figure 1A). A similar trend was seen with the Shannon index (*p*-value = 0.05) (Figure 1B). Beta diversity showed a statistically significant difference between the two groups (p-value = 0.04) (Figure 1C).

Using MAASLIN2 to assess the abundance of specific genera revealed that the abundance of *Prevotella* specifically was lower in the high discrimination group than in the low discrimination group (Figure 1D). Genera belonging to Ruminococcaceae, *Candidatus*, *Methanobrevibacter*, and the order Mollicutes were higher in the high discrimination group than the low discrimination group. A taxonomic plot summarizing major genera is shown in Figure 1E.

### Analysis of predicted metagenomic and transcriptomic profiles reveals potential biomarkers for high discrimination group

**Figure 2:**
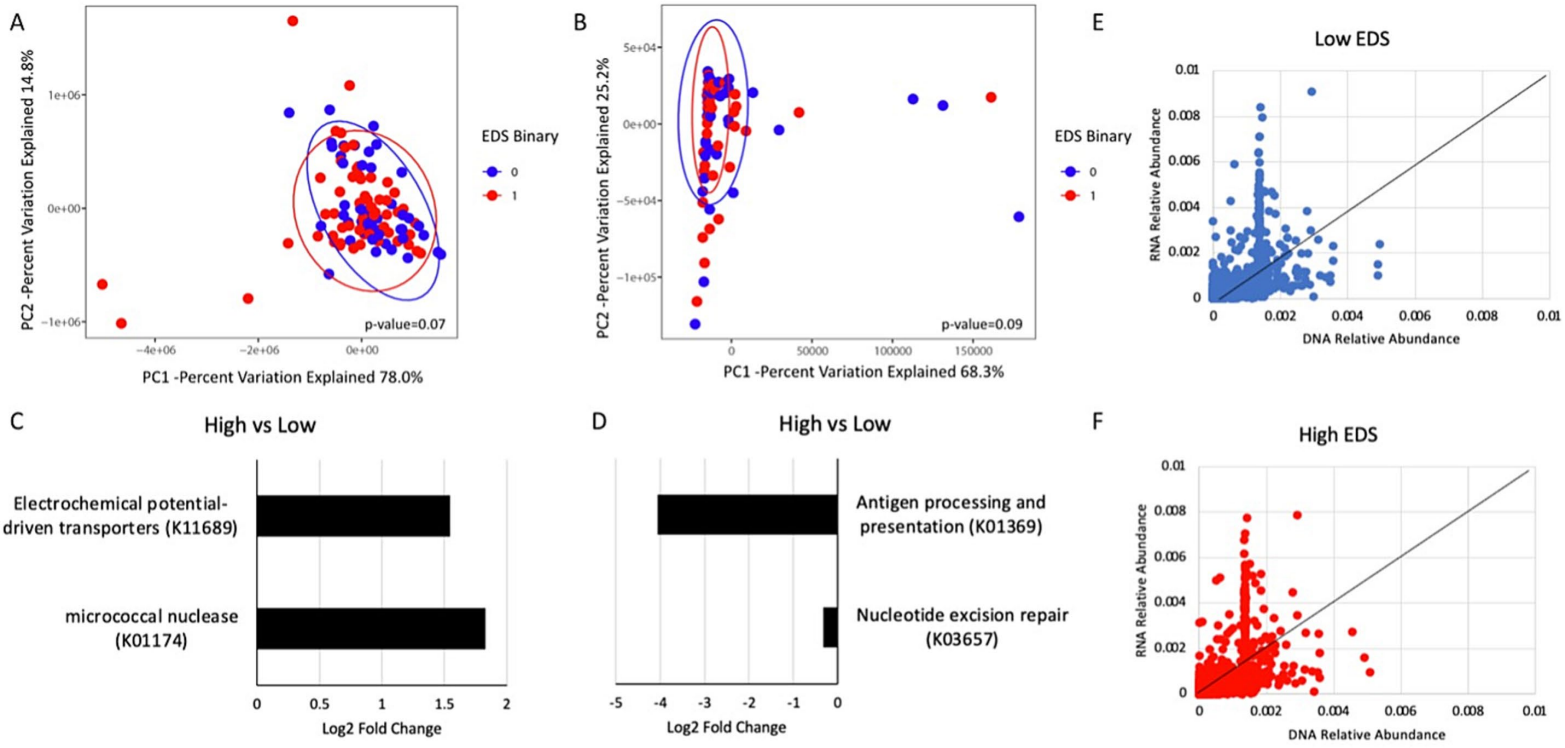
Analysis of predicted metagenomic and transcriptomic profiles in the high and low discrimination groups. **(A)** Principal component analysis plot of predicted metagenomic data and **(B)** transcriptomic data by discrimination. **(C)** DESeq2 analysis showing differential abundant predicted metagenes in the high discrimination group compared to the low discrimination group. **(D)** DESeq2 analysis showing differential abundant transcripts between the high discrimination group and the low discrimination group. **(E)** DNA relative abundance to RNA relative abundance of each gene in the participants with low discrimination and **(F)** high discrimination.

Both the predicted metagenome and transcriptomics show no significant differences between the high and low discrimination groups based on principal component analysis (Figures 2A,B). Differential abundance testing of the predicted metagenome showed that there were higher amounts of electrochemical potential-driven transporters (K11689)—proteins that rely on electrochemical gradients to facilitate secondary active transport—and micrococcal nuclease (K01174)—enzymes that cleave regions between nucleosomes—in the high discrimination group than in the low discrimination group (Figure 2C). Differential abundance testing of the transcriptomic data showed less antigen processing and presentation (K01369) and nucleotide excision repair (K03657) in the high discrimination group than in the low discrimination group (Figure 2D). Generally, the pattern of RNA relative abundances to the DNA relative abundance between the high and low discrimination groups was similar but not identical (Figures 2E,F).

**Figure 3:**
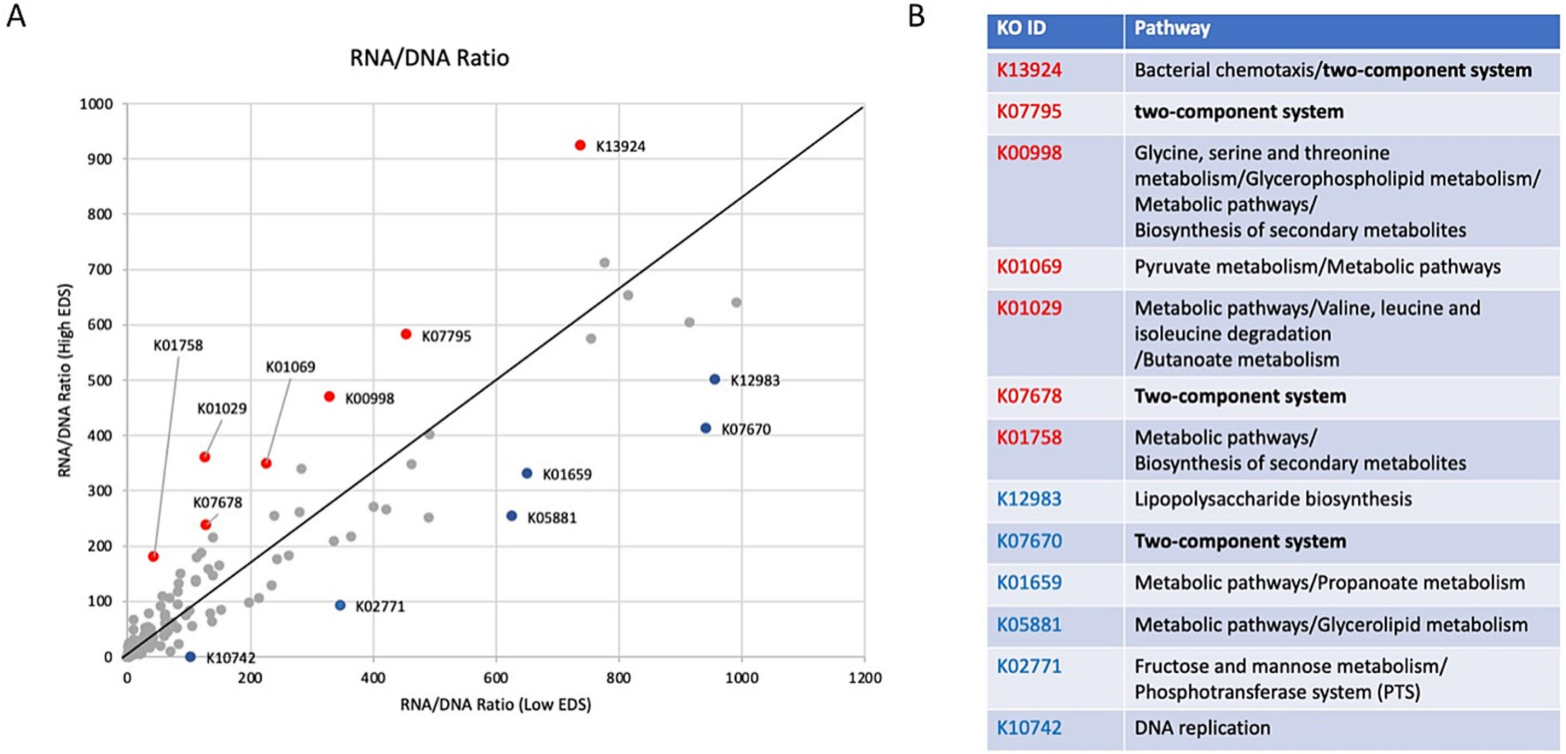
Comparative analysis of RNA-to-DNA ratios and functional descriptions of discrimination-associated genes. **(A)** Graphical comparison of RNA-to-DNA ratios between the low and high discrimination groups. The genes above the line indicate higher transcription rates than those with high discrimination levels, while those below the line represent higher transcription levels in the low discrimination group. Of those genes, the ones highlighted in red and blue represent transcription rates two standard deviations greater than the baseline in the high and low discrimination groups. **(B)** Descriptions of functions for genes indicated to be significantly different between the high and low discrimination groups in figure.

A closer analysis of gene transcription relative to DNA content in participants from the low and high discrimination groups revealed that several genes appear to be transcribed at significantly different levels between the two groups (Figure 3A). Seven genes—K13924 (bacterial chemotaxis), K07795 (two-component system), K00998 (biosynthesis and metabolic pathways), K01069 (pyruvate metabolism), K01029 (metabolic pathways), K07678 (two-component system), and K01758 (biosynthesis and metabolic pathways)—had higher transcription rates in individuals with high discrimination levels. Six genes—K12983 (lipopolysaccharide biosynthesis), K07670 (two-component system), K01659 (metabolic pathways), K05881 (metabolic pathways), K02771 (fructose and mannose metabolism), and K10742 (DNA replication)—had lower transcription rates in individuals with high discrimination levels.

In general, the genes with the most significant changes were associated with environmental sensing (two-component system) and metabolic pathways (Figure 3B).

### Prediction of discrimination status using a multi-omic approach

**Figure 4:**
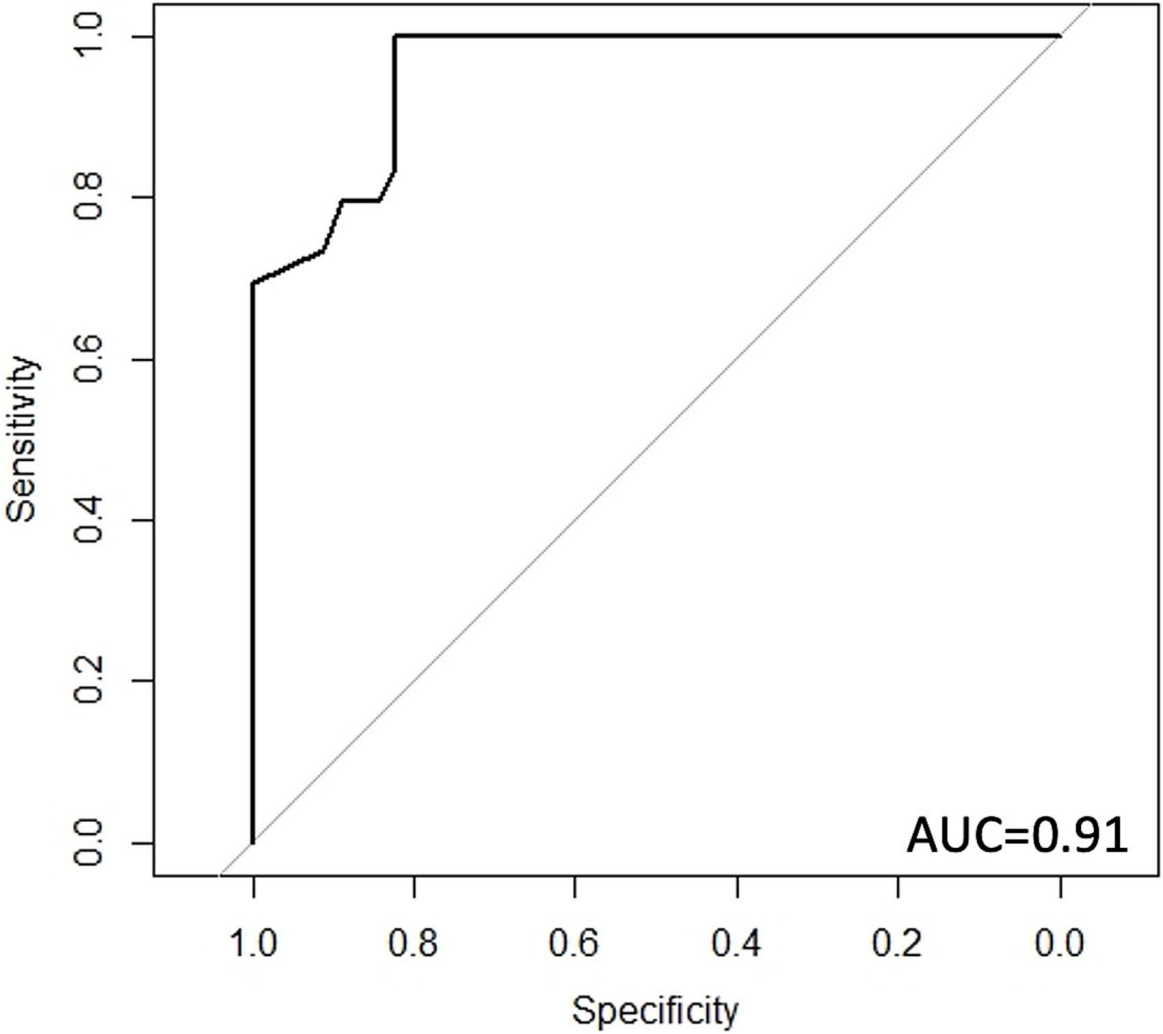
Receiver operating characteristic (ROC) curve for discrimination status classification. ROC curve for random forest classifier using microbiome data, predicted metagenomic data, and transcriptomic data. AUC: area under the curve.

Using a random forest classifier, we showed that a dataset based on the gut microbiome composition, metagenome, and transcriptome could accurately identify participants in the cohort as either in the high or low discrimination group. The model’s receiver operating characteristic (ROC) curve demonstrated a high accuracy, with an area under the curve (AUC) of 0.91 (Figure 4). A model with only microbiome data had an AUC of 0.79, a model with only predicted metagenome data had an AUC of 0.89, and a model with only transcriptomic data had an AUC of 0.86.

## Discussion

Discrimination is associated with significant changes in the gut microbiome profile affecting both the composition of the gut microbiome and its gene expression. A model that encompasses microbiome, metagenomic, and transcriptomic data has a high accuracy for classifying those with high discrimination.

### Discrimination impacts psychological and physical symptoms

Discrimination has been increasingly recognized as a psychosocial stressor with implications for health outcomes (Sawyer et al., 2012). In our study, individuals in the high discrimination group not only exhibited significantly higher levels of adverse psychological traits, including depression, anxiety, and perceived stress, but also reported higher occasions of life adversity and neuroticism. The high discrimination group also had poorer scores overall in physical and mental health. These findings align with existing literature that highlights the deleterious effects of stressors such as discrimination on physical and mental health (Sawyer et al., 2012; Brandt et al., 2022). For example, past studies have linked discrimination to a higher incidence of anxiety and obesity (Chang et al., 2018; MacIntyre et al., 2023; Panza et al., 2019).

The association between visceral sensitivity and discrimination specifically reflects the relationship between psychological stress and physiological response. Visceral sensitivity is the experience of discomfort in an individual’s internal organs (Zhou and Verne, 2011). Chronic stressors, such as discrimination, are considered capable of dysregulating the body’s HPA axis and autonomic nervous system—both critical to the human stress response (Herman et al., 2016; Stephens and Wand, 2012). In our study, the high discrimination group reported heightened visceral sensitivity compared to the low discrimination group. Such physiological changes may contribute to compromised gastrointestinal function.

### Discrimination impacts the gut microbiome

Analyses of the gut microbiome revealed significant differences between the high and low discrimination groups, indicating the potential role of discrimination on the composition of the gut microbiome. One of the most notable observations from these analyses was that the median value for the Chao1 richness estimator was higher for the high discrimination group than for the low discrimination group, suggesting that the high discrimination group has greater species richness. Alpha diversity is used as a common indicator for assessing gut microbiota health and can be closely associated with disease status (Gong et al., 2016). While the exact relationship between higher species richness and inflammation still remains unclear, several studies imply that a higher alpha diversity is considered a biomarker for high-stress environments (Kelly et al., 2021). In this context, a higher Chao1 index implies higher species richness as a response to environmental stressors that can be associated with discriminatory experiences. Taxonomic analysis of the gut microbiome showed that the most notable difference was a decrease in the abundance of *Prevotella* in individuals experiencing high levels of discrimination. *Prevotella* species are associated with the production of short-chain fatty acids and the stimulation of regulatory T-cell production by the immune system. These regulatory T cells play a significant role in suppressing excessive inflammation and maintaining immune homeostasis (Larsen, 2017). *Prevotella* is also linked to various metabolic processes, suggesting that its alteration in individuals with high discrimination may disrupt the host’s health more broadly.

From the microbial gene expression analysis, the differential expression of environmental sensing genes in the high discrimination group is suggestive of an adaptive response by the microbiome to maintain homeostasis in a high-stress environment (Karl et al., 2018; Wu and Wu, 2012). The high discrimination group had less antigen processing and presentation, indicating a weaker host immune response, and less expression of nucleotide excision repair, indicating a potentially weakened DNA damage response (Cheong and Nagel, 2022). The high discrimination group also had significantly higher amounts of micrococcal nuclease (K01174). Micrococcal nuclease catalyzes the hydrolysis of nucleic acids (Alexander et al., 1961). An increased abundance of micrococcal nucleases in the high discrimination group may reflect a greater need for DNA repair or nucleic acid turnover. This enhanced nuclease activity is potentially indicative of a greater need for DNA repair mechanisms due to discriminatory stressors that induce inflammation, thereby increasing the likelihood of DNA damage (Nastasi et al., 2020).

### Prediction of discrimination status

The multi-omic approach, one that combines metagenomic, transcriptomic, and microbiome data, reflected high accuracy in identifying the discrimination status of participants in the trained cohort. This implies that these changes in the gut microbiome are highly linked to discrimination status. While this study has many strengths, such as the use of transcriptomic analysis and comprehensive microbiome analysis, there are several limitations. Because of the sample size, we were unable to split the cohort into a training and validation cohort. While the model was internally validated using k-fold cross-validation, an external cohort will be needed to validate these results. Furthermore, the cross-sectional nature of this study cannot imply causality. Longitudinal studies will be needed to examine the effects of early and late-stage discrimination on the effects of microbiome development.

### Conclusion and clinical implications

Overall, the findings in this study confirm that there is an association between the gut microbiome and its gene expression to discrimination status. The trends revealed in this study emphasize the need for further research to identify the exact mechanisms underlying these relationships. This study provides an initial step toward understanding how inequality manifests into a whole-body experience. Empowering individuals with knowledge about the potential impact of discrimination on their gut microbiome and, more largely, their health could aid in promoting preventative stress and health management strategies. Individuals coping with the physiological and psychological consequences of discrimination could also be supported by actively modulating their gut microbiota through dietary and probiotic interventions tailored to their individual microbiome profiles. By gaining an understanding of how discriminatory experiences shape the microbiome, we can develop targeted interventions that aim to mitigate health inequities as a result of discriminatory experiences.

## Data Availability

Original datasets are available in a publicly accessible repository: 10.5281/zenodo.13921371.
